# Fine tuning of the default depth and rate of ablation of the epithelium in customized trans-epithelial one-step superficial refractive excimer laser ablation

**DOI:** 10.1186/s40662-019-0159-9

**Published:** 2019-12-03

**Authors:** Michael Goggin, Peter Stewart, Vikija Andersons, Giuseppe Criscenti

**Affiliations:** 1 South Australian Institute of Ophthalmology, University of Adelaide, Adelaide, Australia; 2 Brisbane Laser Sight Clinic, Brisbane, Australia; 3 Ashford Advanced Eye Care, Adelaide, Australia; 4 iVis Technologies, Taranto, Italy

**Keywords:** Transepithelial photorefractive keratectomy, Epithelial ablation, Epithelial thickness refinement, Stromal ablation rate refinement

## Abstract

**Purpose:**

To fine tune the default depth and rate of ablation of the epithelium in cTen™ customized trans-epithelial one-step superficial refractive surgery by the comparison between the attempted post-operative ideal corneal shape and the achieved corneal shape.

**Methods:**

88 consecutive eyes in 64 patients undergoing trans-epithelial superficial excimer ablation using the iVis laser Suite for either myopic/astigmatic or hyperopic/astigmatic refractive error. Each patient had at least 3 months of post-operative follow-up. Topographic examination of all eyes was carried out pre-operatively and at least 3 months post-operatively using the Precisio™ surgical topographer. The comparison of these two measurements yielded values for depth, volumes and rates of ablated corneal tissue. By determining the different ablation rates of stroma and epithelium, a refinement of the depth of epithelium to be removed and a refinement of the stromal ablation were calculated.

The mathematical model was applied on each one of the 88 clinical cases and the parameters for the fine tuning of the default depth and rate of ablation of the epithelium were determined using the least squares method.

**Results:**

The calculated pure stromal ablation rate was less than the average epithelium/stroma ablation rate used in planning the treatments by a factor of 0.96. The epithelial thickness predefined ablation assumption used to plan removal of the epithelium was adjusted considering the measured ablation and a radial adjustment function established for the fine tuning of the laser radial efficiency and allowing for the normal thickening of the epithelium in the peripheral cornea. From a clinical point of view, this methodology produces an improvement of the efficacy and a reduction of the variance of the clinical results.

**Conclusion:**

Comparison of accurately measured pre and postoperative topographies yields accurately established ablation rates of stroma and epithelium in trans-epithelial one step superficial ablation.

## Background

Photorefractive keratectomy (PRK) is commonly performed in conjunction with manual removal of the central 6 to 9 mm of corneal epithelium by one of several methods. These include mechanical debridement with a spatula or similar instrument, an automated brush or with a keratome, sometimes facilitated by prior exposure of the epithelium to alcohol. The area thus denuded of epithelium is of necessity larger than the area of stromal ablation and invariably has an irregular edge [1–3].

With the manual removal of the epithelium, significant differences in UDVA, pain score, level of haze and complete epithelial healing time in the early postoperative period were detected by comparison with the laser removal approach [3].

In the last couple of decades, the possibility to remove automatically the corneal epithelium in a single-step procedure using a laser source represented a new alternative for laser refractive error correction [4].

The iRes excimer laser (iVisTechnologies, Taranto, Italy) performs PRK by ablating the epithelium to a pre-set default constant depth and ablating the customized stromal depth, all in a single surgical step. This technique specifically restricts the epithelial removal exactly to fit the chosen area of stromal ablation and provides a regular curvilinear post-operative epithelial edge [5–10].

However, to achieve a successful epithelial ablation, an assumption of epithelial thickness based on the manufacturer’s laboratory measurements is made. A proprietary pre-set epithelial thickness is used for this technique which assumes thinner epithelium centrally than peripherally. However, the use of a default pre-set epithelial depth might induce an error in stromal ablation if the assumption is not correct.

The first aim of this study is to establish whether the default pre-set epithelial depth assumption is correct by comparison of the attempted ablations with that achieved, using a topographic measure of the ablation of both stroma and epithelium combined. The second aim of this study is to establish if the assumed epithelium and stroma average tissue ablation rate is correct.

## Methods

The diagnostic analysis and the surgical treatments described in this study were performed with iVis Suite platform (iVis Technologies, Taranto, Italy) which include the surgical topographer Precisio™, the dynamic pupilometer pMetrics™, the Corneal Interactive Programmed Topographic Ablation CIPTA® software, the 1 kHz excimer laser IRES™ and the iVerify™ statistical web application. Precisio™ is a surgical topographer, designed for customized refractive surgery, equipped with a dedicated eye-tracker system that can detect the anterior corneal shape with a repeatability lower than 3 μm. The software medical device CIPTA® is designed to plan the customized tissue volume to be ablated defining the ideal corneal shape needed to achieve the desired correction and consequently calculate the volume of ablation as the difference between the Precisio™ detected anterior corneal shape and the ideal corneal shape.

In this study, 88 normal eyes in 64 patients underwent one-step customized trans-epithelial ablations using the iRES excimer laser. The surgeries were performed in two private refractive surgery centers in Australia (Brisbane Laser Sight Clinic, Brisbane, and Ashford Advanced Eye Care, Adelaide).

The patients included in this study met the following inclusion criteria:

- Over the age of 18
- With a refractive defect of:
  - ◦ Myopia
  - ◦ Hyperopia
  - ◦ Simple and compound astigmatism
  - ◦ Mixed astigmatism
- Planned and executed customized trans-epithelial one step superficial keratorefractive surgery cTen™ with a default tissue ablation rate and a default constant preset epithelial thickness, thinner centrally than peripherally
- Having a repeatable Precisio™ topographer exam acquired either for the pre-operative planning exam, as well as for the 3 months follow-up post-operative exam, with valid maps covering a corneal diameter of 8 mm or greater

The repeatability analysis of the acquired exam is automatically performed by the Precisio™ surgical topographer. It compares the first topographic examination with the second one that is taken immediately after the first as a routine procedure. The difference between the first and second topographic measurements on each mapped location is accepted only if it is 3 μm or less throughout a minimum 6 mm central corneal zone. In addition, with iris and pupil registration at each step with this device (the pre-operative examination, the surgery and the post-operative examination), the X, Y and rotational location of topographic change from the ablation can be established.

The achieved ablations are measured as the difference between the anterior pre-operative corneal shape and the anterior post-operative shape which are both detected using the Precisio™ surgical topographer.

The attempted anterior post-operative shapes are determined as the difference between the anterior pre-operative corneal shapes detected by the Precisio™ topographer and the executed customized ablation profiles planned with CIPTA™ software.

The comparison between the attempted ablation and the achieved ablation is executed, on a point by point basis, in a radial direction, by means of the iVerify™ statistical application which allows the determination of the effective radial ablation rate analyzing the point-by-point local difference between the attempted ablation and the achieved ablation over a large database.

Surgeries were performed using the iRES™ excimer laser which ablates at a tunable repetition rate up to 1000 Hz but adjusted to a constant frequency beam delivery on the cornea equal to 5 Hz/mm^2^ to avoid thermal effects and achieve smooth profiles. During the whole treatment, the laser spot is delivered with a constant fluence of energy per unit of time.

The trans-epithelial approach described above was delivered in one step with a customized stromal ablation pattern based on the individual patient’s refractive error and tailored to their topographic pattern. The customized area and outline of the epithelial ablation correspond to the area and outline of the stromal refractive ablation. Considering this approach, not all ablations are circular and all are “customized” so the effect of the ablation on the eye to be treated is calculated in order to have optimal edge profiles.

The post-operatively established changes in corneal shape were compared with the predicted ablation depths. The comparison was done in the center of the ablation as well as at varying radial distances (designated “j”) from the center of the ablation (for mapping purposes designated as location “0;0”) up to the radius of 5 mm from the center, where j = radial distances of 0.5 mm, 1.0 mm, 1.5 mm, 2.0 mm, 2.5 mm, 3.0 mm, 3.5 mm, 4.0 mm, 4.5 mm and 5.0 mm. These data were collected on a Cartesian grid of 100 μm at each one of the following annuli; 0.5 mm +/− 100 μm, 1.0 mm+/− 100 μm, 1.5 mm+/− 100 μm, 2.0 mm+/− 100 μm, 2.5 mm+/− 100 μm, 3.0 mm+/− 100 μm, 3.5 mm+/− 100 μm, 4.0 mm+/− 100 μm, 4.5 mm+/− 100 μm and 5.0 mm+/− 100 μm. This covers a possible ablated zone of up to 10 mm. Mean values within these areas were determined and compared (attempted versus achieved).

The best approximation of the achieved ablation depth thus derived at the center of the ablation (0;0) was compared with the attempted ablation calculated in the center of the ablation (0;0), according to the following function:

R_i_ = xEs_i_+(y-1)Ep_,_

where

i = 1 … n, n = treated eye number;

R_i_ is the achieved value of ablation depth including the epithelium for each treated eye;

Es_i_ is the expected value of the ablation depth of the stroma for each treated eye;

Ep is the default epithelial depth value used to ablate the epithelium;

x is the correction constant to be defined for the stromal ablation;

y is the correction constant to be defined for the epithelium ablation;

(y-1)Ep is the corrected constant of the total epithelium ablation depth;

x and (y-1)Ep are calculated applying the least squares method.

The determination of the coefficients of the above function provided the best corrective x constant for the stromal ablation rate. The proprietary IRES laser energy factor used in the treatments (which is an average of stromal and epithelial ablation rates) was multiplied by this constant to define the new pure stromal ablation rate. The best corrected (y-1)Ep constant of the ablation depth for the epithelium was also similarly established and applied to the default constant value at the center of the ablation (0;0).

The determination of the (y-1)Ep_j_ values at j radial distance from the center of the ablation (0;0) up to the radius of 5 mm from the center (the 10 mm maximum ablation zone of the device) was obtained by the best approximation of the mean achieved ablation depth versus the expect ablation depth calculated according to the following function:

R_i_ = xEs_ij_+ (y-1)Epi_j_.

where

i = 1 … n, n = treated eye number;

j = radial distance (0.5 mm, 1 mm, 1.5 mm, 2 mm, 2.5 mm, 3 mm, 3.5 mm, 4 mm, 4.5 mm and 5 mm);

R_i_ is the mean achieved value of ablation depth including the epithelium for each treated eye at j distance from the center of the ablation;

Es_i_ is the expected value of the stromal ablation depth for each treated eye at j distance from the center of the ablation;

x is the correction constant as defined for the stromal ablation above;

(y-1)Ep_j_ is the corrected constant of total epithelium ablation depth defined for each j radial distance from the center of the ablation (0;0).

The determination of the (y-1)Ep_j_ coefficients provided the data set for each j radial distance from 0.5 mm radius up to the radius of 5 mm from the center of the ablation (0;0), again covering the maximum ablation zone of 10 mm.

Finally, the function that describes the epithelial thickness used to ablate the epithelium in one step superficial refractive surgery, defined as corrected f(radial distance), is obtained by fitting of the (y-1)Ep_j_ values with a proprietary second order function related to the distance from the center of the ablation (0;0) up to 5 mm. This approach allows the gradual radial thickening of the epithelium in normal corneas.

### Statistical analysis

The measure of the Least Squares approximation goodness was assessed by the determination coefficient (*R*^*2*^ ≥ 50%) and the correlation coefficient of Bravais-Pearson (*R* > 70%). The R correlation coefficient was calculated to validate the hypothesis of a linear interdependence between the real ablation data set and the attempted ablation data set through the x and (y-1)Ep coefficients. It ranges in value from − 1 to + 1, indicating perfect negative correlation at − 1, absence of correlation at zero, and perfect positive correlation at + 1. The coefficient of correlation R should be greater than 0.70 to show a good linear correlation. The coefficient of determination, denoted by R [2], was calculated to measure the percentage of variability of R in function of the variability of E. It ranges in value from 0 to 100%. The coefficient of determination R^2^ should be greater than or equal to 50%.

After the determination of the x and (y-1)Epij values, the weighted mean ratios of Ri values vs. (xEsii + (y-1)Epij) values are calculated at each radial distance j from the center of the ablation up to a distance of 3 mm from the center. The percent mean error E% and the precision D, expressed as follows, should meet the following values for each x and (y-1)Epij:

$$ E\%=\left( Weighted\kern0.17em mean\left(\frac{R_i}{\left( xE{s}_i+\left(y-1\right)E{p}_{ij}\right)}-1\right)\right)\ast 100\le 30\% $$

D ≤ 5%.

To assess the reliability of the obtained results, according to the Gaussian assumption, the 95% confidence interval (*CI*) for the mean *M* of the ratios between observed data and estimated data was considered at all radial distances and the corresponding precision *D* was calculated and analyzed:

CI = M ± k*SE.

D = k*SE*100 ≤ 5%,

where *k = 1.96* is the *α/2* quantile of the standard normal distribution with *α = 0.05* and *SE* is the standard error equal to the standard deviation divided by the square root of the treated eye number: $\mathrm{SD}/\sqrt{\mathrm{n}}$.

## Results

This study evaluated 88 eyes of 64 patients (51 right eyes and 37 left eyes). Demographic information of the population are summarized in Table 1.

**Table 1:** Demographic information of the population used in this study

Demographics		Sites				Site 1 + Site 2	
		1		2			
Number of eyes & subjects		35 eyes of 24 enrolled subjects		53 eyes of 40 enrolled subjects		88 eyes of 64 enrolled subjects	
		*n*	%	*n*	%	*n*	%
Gender	Male	7	29.2	12	30	19	29.7
	Female	17	70.8	28	70	45	70.3
Surgical eye	Right	20	57.1	31	58.5	51	58
	Left	15	42.9	22	41.5	37	42
Age (in years)	Mean (SD)	37.9 (13.4)		46.2 (13.6)		42.8 (14.2)	
	Minimum	21.0		22.0		21.0	
	Maximum	68.0		70.0		70.0	

The mean follow-up time was 3.96 ± 1.15 months (range: 3 to 7 months). The mean spherical equivalent was − 2.08 ± 2.17 D (range: − 7.30 to 2.58 D). Approximation with the Least Squares method of the achieved ablation depth determined at each distance “j” from the center of the ablation (0;0) vs. the expected ablation calculated at each distance “j” from the center of the ablation (0;0), for each one of the 88 following functions is as follows:

R_ij_ = xEs_ij_ + yEpij where i = 1 … n, n = 88 j = 0, 0.5, 1 …. 5;

The following results were obtained (Table 2).

**Table 2:** Obtained values for the corrective constants x and (y-1)Ep, the parameters for the goodness of fit and the parameters for the data reliability assuming the model described in methods section

x	(y-1)Ep	*R* (%)	*R*^2^ (%)	*E*_%_ (%)	*D* (%)
0.96	10.459	96.08	92.31	−0.58	3.63

The correction constant x for pure stromal ablation versus the average ablation rate which includes both epithelium and stroma is equal to 0.96. This indicates that the previous default value was under-ablating the corneal stroma by approximately 4.0%. It can be observed that the correlation coefficient R is greater than 70%, so there is a linear dependence between real and attempted ablation depths. Moreover, the coefficient of determination R^2^ is greater than 50%, thus the assumed model is correct. With respect to data reliability, the inequality E% ≤ 30% is satisfied as well as the inequality D ≤ 5%.

According to the results shown in the preceding section, the corrective constant x is set at 0.96 with the model described in the previous section to evaluate the corrective constants (y-1)Epij where j is the radial distance from the center of the ablation and it ranges from 0 mm to 3 mm with a step of 0.5 mm.

In Table 3, the values obtained for the corrective constants (y-1)Epij and the corresponding reliability parameters are shown.

**Table 3:** Corrective constants (y-1)Epij values and corresponding reliability parameters

	Radial Distance from the center of the ablation (in mm)						
	0	0.5	1	1.5	2	2.5	3
(y-1)Epij	10.361	10.100	9.293	8.036	6.549	5.481	5.296
*E*_%_ (%)	−0.62	−0.66	−0.55	− 0.25	0.29	1.16	3.16
*D* (%)	3.63	3.67	3.83	4.09	4.43	4.69	5.67

**Fig. 1:**
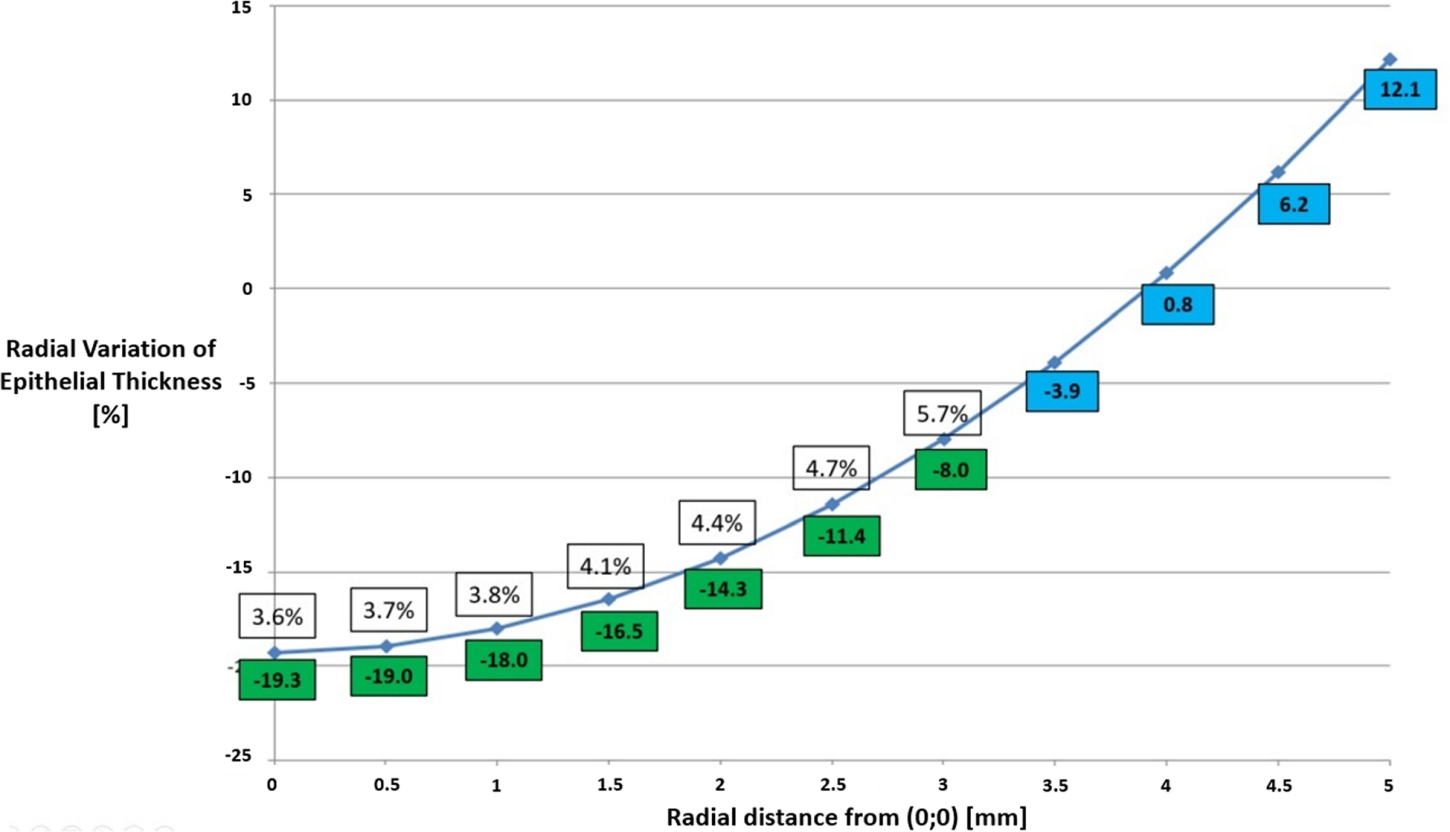
Percentage radial variation of the epithelium thickness with respect to the default constant value. The post-operative measured data (green), the extrapolated data (blue) and the precision values D(%) (squares) are shown

As shown in Fig. 1, the percentage radial thickening of the epithelium with respect to the default constant value ranged from − 19.3 to + 12.1%. In particular, a reduction of 19.3% of the default constant value was calculated in the central zone. At a radius of approximately 4 mm, the default constant value was similar to the calculated one (0.8%) while an increase of 12.1% of the constant default value was found to be necessary at the 5 mm radius annulus. These data were calculated depending on the effective solutions, using the least squares method, of the R_ij_ = xEs_ij_ + yEpij equations up to 2.5 mm from the center, where the observed data were reliable according to the precision previously defined (less than 5%). The data between 3.0 and 5.0 mm, having a precision greater than 5%, were extrapolated from the data.

## Discussion

In this study, a method is described to fine tune the default depth and rate of ablation of the epithelium in cTen™ customized trans-epithelial one-step superficial refractive surgery by comparing the attempted post-operative ideal corneal shape and the achieved corneal shape. The cTen surgical approach has been demonstrated to have high stability at 1 month postoperative follow up [11]. Each patient had at least 3 months of post-operative follow-up and the mean follow-up time was 3.96 ± 1.15 months (range: 3 to 7 months).

The calculated pure stromal ablation rate was less than the average epithelium/stroma ablation rate used in planning the treatments by a factor of 0.96. The epithelial thickness assumption used to plan removal of the epithelium was adjusted on the basis of the measured ablation and a radial adjustment function established. This radial adjustment was calculated from the observed normal radial increase in epithelial thickness toward the periphery and was used to fine tune the laser’s radial efficiency.

In order to perform trans-epithelial refractive laser ablation, the exact knowledge of the epithelial thickness and the ablation rate of the epithelium and of the stroma is mandatory. Excess ablation wastes stromal tissue and variations in epithelial thickness of the ablated area may influence refractive outcome [1, 2]. Under-estimation of these parameters may contribute to a reduction of the optical zone while an incorrect evaluation of the epithelium profile to be ablated may affect the aspherical treatment outcome due to the different ablation rate and, consequently, different ablation profile. Under- or over-estimation of the rate of ablation either of the epithelium or stromal tissue will also influence the refractive outcome.

The method of defining the customized ideal shape needed to optimize the quality of vision, instead of the most common approach of printing a refractive lens onto the cornea, allows for the determination of the effectively achieved ablation volume, inclusive of post-operative re-epithelization process and induced biomechanical changes. In fact, the differences between the attempted and the achieved volume of ablation may be effectively determined by means of the comparison between the attempted ideal corneal shape and the achieved post-operative corneal shape. By comparison of the attempted vs. achieved ablation patterns over a large database, using accurate topographic information, the assumed values of these parameters can be refined. Prior to refinement, the epithelial thickness was assumed to have a proprietary default profile across the whole ablated area (thinner centrally than peripherally) and the stromal ablation rate equal to the epithelial ablation rate. Examination of the true stromal ablation rate and true epithelial thickness suggests a refined pure stromal ablation rate increment of 4.0% on the rate used for the treatments in the study. A radial variation of the epithelial thickness to be ablated is suggested with a reduction of the assumed epithelial thickness equal to 19.3% in the center of the ablated area and an increase of the assumed epithelial thickness equal to 12.1% at the 5 mm radius (the maximum area that can be ablated with the iRES excimer laser). It should be pointed out that the apparent epithelial thickness established here is in terms of ablation rates. The used method includes the effect of the radial efficiency of the laser and the effect on corneal thickness of corneal epithelial regrowth and healing which may not return to an epithelium that is exactly the same as prior to surgery. Consequently, it may be different to epithelial thickness measured by optical systems but is a measure of “functional” epithelial thickness.

In common with all other laser systems, an ablation rate of corneal tissue is established by the manufacturer’s laboratory measurements and applied in treatment planning. This proprietary average includes ablation of both stroma and epithelial tissue. Comparison of attempted ablations versus achieved ones, by topographic measures, can be also used to refine this value and to refine the value assumed for epithelial thickness.

Trans-epithelial one step refractive excimer laser ablation cTen™ is comparable in terms of outcomes to traditional alcohol-assisted or manual removal of epithelium [12–15]. Further, there is some evidence showing that it may provide better visual outcome in eyes with low to moderate myopia when compared with LASIK, LASEK and manual epithelial removal PRK [16]. With the very high ablation rate of the IRES excimer laser (1000 Hz), this procedure is performed rapidly as the excimer laser removes the epithelium and ablates the stroma in one single step [12]. There is less post-operative pain and faster epithelial healing probably because a precise zone of epithelium is removed only where the stromal ablation will take place [14, 17, 18]. Moreover, trans-epithelial single step superficial refractive surgery has significantly less post-operative corneal haze at 1, 3, 6 and 12 months when compared to conventional PRK [18].

Myopic and hyperopic ablations lead to opposite morphological changes and cause opposite postoperative epithelial and biomechanical changes. This is a possible drawback of our technique but accurate pre- and post-operative topographic information in conjunction with the Least Squares method, calculated for individual eyes, will allow one to limit this possible source of error without the need to study different ablation types.

Prior to this study, the iRES excimer laser ablated the epithelium to a default pre-defined depth (thinner centrally than peripherally). Refinement of the epithelial thickness assumption, both centrally and peripherally, allows more accurate ablation depth planning and the ablation of only the stromal tissue needed for refractive correction. In addition, an accurate estimation of stromal ablation rates allows for a better treatment plan and assists in achieving more accurate outcome, improvement of the efficacy and reduction of the variance of the clinical results.

Ongoing audit of these data and precise epithelial mapping will further contribute to better outcome in the future.

## Conclusion

The comparison of accurately measured pre- and post-operative topographies yields accurately established ablation rates of stroma and epithelium in trans-epithelial one step superficial ablation. Modification of the assumed rates to the rates thus established may lead to more accurate topographic and consequently, more accurate refractive outcome with an improvement of the efficacy and a reduction of the variance of the clinical results. The analytical techniques used in this study are applicable to further data examining ablation rates as they may vary with patient age or disease state or with different ablation techniques.

## Data Availability

Not applicable.
